# Amino Acid Profile and Protein Quality Assessment of Macroalgae Produced in an Integrated Multi-Trophic Aquaculture System

**DOI:** 10.3390/foods9101382

**Published:** 2020-09-29

**Authors:** Marlene Machado, Susana Machado, Filipa B. Pimentel, Victor Freitas, Rita C. Alves, M. Beatriz P. P. Oliveira

**Affiliations:** 1 LAQV, REQUIMTE, Department of Chemical Sciences, Faculty of Pharmacy, University of Porto, 4050-313 Porto, Portugal; marlenemachado753@gmail.com (M.M.); smachado@ff.up.pt (S.M.); beatoliv@ff.up.pt (M.B.P.P.O.); 2 LAQV, REQUIMTE, Department of Chemical and Biochemistry, Faculty of Sciences, University of Porto, 4169-007 Porto, Portugal; vfreitas@fc.up.pt

**Keywords:** seaweeds, conchocelis, amino acids, protein quality, integrated multitrophic aquaculture

## Abstract

Seaweeds are a recognized source of bioactive compounds and techno-functional ingredients. However, its protein fraction is still underexplored. The aim of this study was to determine the total and free amino acid profile and protein content of four seaweeds species (*Porphyra dioica, Porphyra umbilicalis,* *Gracilaria vermiculophylla,* and *Ulva rigida*) produced in an integrated multi-trophic aquaculture system, while assessing their protein quality. Samples were submitted to acid and alkaline hydrolysis (total amino acids) and to an aqueous extraction (free amino acids) followed by an automated online derivatization procedure, and analyzed by reverse phase-high performance liquid chromatography. Protein-, non-protein and total-nitrogen were quantified by the Kjeldahl method. Crude and true protein contents were estimated based on the nitrogen and amino acid composition. Protein quality was assessed based on the amino acids profile. *Porphyra* species presented the highest protein content compared to the remaining three seaweed species tested. All samples presented a complete profile of essential amino acids and a high quality protein profile, according to World Health Organization and Food and Agriculture Organization standards. Methionine and tryptophan were the first limiting amino acids in all species. Red species (*Porphyra* and *Gracilaria*) presented high levels of free alanine, glutamic, and aspartic acids. The results highlight the potential of using seaweeds as an alternative and sustainable source of protein and amino acids for human nutrition and industrial food processing.

## 1. Introduction

Seaweeds are a diverse group of marine species that have been part of the human diet for thousands of years, especially in Asian countries. In western countries, however, algae are mainly exploited as a source of techno-functional polysaccharides (carrageenan, agar, and alginates), used as technological aids (such as texturizing and stabilizing agents in foods, for example) [1,2]. Besides, seaweeds may also have agriculture application. The development of novel, sustainable, and eco-friendly solutions is an extremely relevant topic in this field. For instance, some authors have reported that the use of a small quantity of different seaweed liquid extracts efficiently enhanced the in vitro mass propagation of an important crop of *Solanum melongena* L. This suggests the possibility of using it as an alternative to commercially available plant growth regulators that can be toxic and costly when compared with natural extracts [3]. The antimicrobial activity of seaweeds is also often reported. Recently, a study explored the non-bactericidal anti-virulence efficacy of green synthesized silver nanoparticles from *Gelidiella acerosa* against multi-drug resistant *Vibrio* spp. Results were very promising, indicating the possibility of using the above-mentioned algae-derived nanoparticles as an alternative solution for controlling vibriosis in the culture of brine shrimp larvae, with no associated toxicity [4]. Due to the presence of bioactive or phytochemical compounds, seaweeds have been increasingly suggested as “functional foods” or “nutraceuticals,” or in other words, as foods that benefit health beyond their nutritional role [5]. Although the detailed chemical composition of these marine organisms is not fully described yet, they are known to be a rich and sustainable source of macronutrients and micronutrients to the human diet [5,6]. Many macroalgal species, in particular the red seaweeds, have significant protein levels, some of which higher than other protein-rich foods, such as soybean, cereals, eggs, and/or fish [1].

The amino acid composition is essential to determine the protein quality of food in the human diet, particularly to achieve an adequate intake of essential amino acids [6]. Seaweed proteins contain significant amounts of essential amino acids, accounting for almost 50% of their total amino acid composition [7,8]. It should be noted that amino acids have a range of important physiological roles, including regulating food intake, gene expression, protein phosphorylation and cell-to-cell communication, among many others [9]. Furthermore, amino acids are essential precursors for the synthesis of hormones and nitrogenous substances of low molecular weight, each with great biological importance [9]. As the amino acids have specific physiological functions, they can be used to help manage some diseases. For instance, supplementation with methionine can be effective in patients with multiple sclerosis [10]; arginine therapy can have a neuroprotective effect after brain ischemia injury [11]; histidine can improve insulin sensitivity and thus alleviate hyper-insulinemia [12]; supplementation with glycine can help alleviate liver and lung injury [12]; tryptophan is used to improve sleep disorders and depression [9].

The global seaweed industry is estimated to worth more than USD 6 billion per annum, corresponding to approximately 12 million tons per annum in volume. By 2015, the global production reached 30.4 million tons, 29.4 and 1.1 million tons from cultivation and wild harvest, respectively [13].

Aquaculture practices have been growing in response to rising demand for algal biomass. Seaweeds can be easily cultivated on large scales in coastal areas with little or no demand on freshwater resources in production cycles. In addition, their growth rates exceed those of terrestrial plants, spurring the interest for the cultivation of seaweed biomass [14]. The cultivation methods and technologies can be diverse depending on the genus [15]. Integrated multi-trophic aquaculture (IMTA) is one of the methods offering many advantages. The IMTA system allows several species of different trophic levels to be produced/cultivated together, where the by-products of one species are recycled and become a source of nutrients for another [16]. In this way, the IMTA system minimizes the environmental impacts caused by aquaculture (mainly fish farms) while creating eco-efficiency, environmental acceptability, product diversity, competitiveness, and social benefits [16,17]. For example, the combination fed aquaculture species (such as salmon) with inorganic extractive aquaculture species (seaweeds) and/or extractive organic aquaculture species (suspension and deposit) can be used to increase production efficiency and reduce waste [17]. Studies report that production in the IMTA system improves the growth of extractive species when there is a high concentration of nutrients (e.g., areas close to fish farms) [18]. Angell et al. [19] also reported that cultivated seaweeds have a higher protein content compared to wild-harvested seaweeds because the latter grow in environments that are often nutrient-limited, whereas cultivated seaweeds grow in nutrient-rich water from artificial land-based systems.

The Genus *Porphyra* is one of the most important cultivated seaweed in the world, with 1.2 million tons produced in 2015 [13]. Due to their high surface/volume ratio, these species grow and assimilate nutrients rapidly. These attributes suggest that this genus is one of the most promising for bioremediation and cultivation in IMTA systems [20]. *Porphyra* reproduces by both sexual and asexual modes. In sexual reproduction, carpogonia fertilization by spermatia occurs in a gametophytic blade. After fertilization, the carpogonia divides to form packets of spores called carpospores. These grow by unipolar germination to produce the sporophyte called conchocelis that has a filamentous and branched form. Under certain conditions, *conchocelis* filaments develop and produce conchospores, which give rise to a thallus (blades) that completes the life cycle [21,22].

*Ulva* sp. and *Gracilaria* sp. are also known for their high growth rate. *Ulva*, in particular, can yield more than 20 g of dry weight/m^2^/day, one of the highest rates among photosynthetic organisms [7]. *Gracilaria vermiculophylla*, a non-indigenous Asian species naturalized in Ria de Aveiro, Portugal, is known to be highly resistant to several stressful factors, including the absence of light, sedimentation, desiccation, and different nutritional conditions [20]. Due to the particular characteristics of the above-mentioned seaweed species, their production in IMTA systems has been explored [20,23,24].

This study aimed at characterizing the protein profile of four species of seaweeds (*Ulva rigida, Gracilaria vermiculophylla, Porphyra umbilicalis,* and *Porphyra dioica*) produced in an IMTA system. This involved the determination of free and total amino acids composition of the selected seaweeds, including in different life cycle stages of the species *Porphyra dioica* and *Porphyra umbilicalis* (*conchocelis* and adult blades) and the assessment of the protein quality of the samples based on their amino acid score (AAS) and essential amino acid index (EAAI).

## 2. Materials and Methods

### 2.1. Reagents and Standards

Boric acid was acquired from Chem-Lab (Zedelgem, Belgium). HPLC-grade methanol and acetonitrile and sodium azide (99%) were from Honeywell Riedel-de Haën (Seelze, Germany). Hydrochloric acid ≥ 37% and sulfuric acid 96–97% were obtained from Honeywell Fluka (Düsseldorf, Germany). Disodium tetraborate decahydrate (99–103%), disodium hydrogen phosphate anhydrous (≥99%), potassium hydroxide, trichloroacetic acid (≥98%), and Kjeldahl catalyst tablets were from Merck (Darmstadt, Germany). Sodium hydroxide was from LabChem (Loures, Portugal). Borate buffer, o-phthalaldehyde/3-mercaptopropionic acid (OPA/3-MPA), and 9-fluorenylmethyl chloroformate (FMOC) were from Agilent Technologies (Palo Alto, CA, USA). The amino acid kit containing the individual standards (≥99%) of L-alanine, L-arginine hydrochloride, L-asparagine, L-aspartic acid, L-cysteine, L-cystine, L-glutamic acid, L-glutamine, glycine, L-histidine hydrochloride, trans-4-hydroxy-L-proline, L-isoleucine, L-leucine, L-lysine hydrochloride, L-methionine, L-phenylalanine, L-proline, L-serine, L-threonine, L-tryptophan, L-tyrosine, and L-valine and L-norvaline was from Sigma-Aldrich (Darmstadt, Germany). Ultrapure water was obtained from a Seralpur PRO 60 CN and Seradest LFM 20 water purification system (Ransbach-Baumbach, Germany).

### 2.2. Algal Biomass

The algal biomass was provided by ALGAplus Ltd. (lhavo, Portugal) from a production site at Ria de Aveiro, Portugal (40º36′43” N, 8º40′43” W). Samples of *Ulva rigida*, *Gracilaria vermiculophylla*, *Porphyra umbilicalis,* and *Porphyra dioica* were produced in an IMTA system and supplied dry. The *conchocelis* phase of *Porphyra dioica* and *Porphyra umbilicalis* were cultivated in an indoor nursery, under controlled conditions. The collected biomass was washed with fresh water and kept at −20 °C prior to freeze-drying (48 h, −80 °C, 0.015 mbar) in a Telstar Cryodos-80 freeze dryer (Telstar, Terrassa, Spain). The samples were then milled (Thermomix^®^, TM5, Vorwerk, Germany) and stored in vacuum-sealed bags, and stored protected from light until analysis.

### 2.3. Amino Acid Composition Analysis

#### 2.3.1. Total Amino Acids Extraction

The total amino acids were determined according to the protocol described by Machado et al. [25] with minor modifications. Briefly, 150 mg of sample were weighed and mixed with 3 mL of 6 M HCl in a screw-cap tube. The reaction tubes were flushed under an N2 stream during 5 min in order to minimize oxidation and immediately closed prior to being placed in a Thermo block (SBH130D/3, Stuart, Stafford, UK) at 110 °C for 24 h. The hydrolyzed samples were centrifuged (3000 rpm, 10 min, Megafuge 16 centrifuge, Heraeus, Germany) and 50 µL of supernatant was collected and neutralized with 940 µL of borate buffer (pH 10.2). The internal standard (10 µL norvaline 2 mg/mL) was added to each sample. The homogenized mixtures were centrifuged for 10 min at 13,000 rpm (Biofuge *pico* Heraeus, Hanau, Germany) and supernatants were finally transferred into injection vials for amino acid analysis. Alkaline hydrolysis was used to determine the content of tryptophan as it degrades under acidic conditions. Alkaline hydrolysis was performed using a similar protocol, with modifications in the following steps: 150 mg of each sample were weighed and mixed with 3 mL of 4 M KOH; the tubes containing the samples were put in a Thermo block (4 h, 110 °C); following hydrolysis, the samples were neutralized with 0.1 M HCl. The extractions were performed in triplicate for each sample.

#### 2.3.2. Free Amino Acids Extraction

The extraction of free amino acids was based on the study by Machado et al. [25]. Briefly, samples were prepared by dispersing the milled sample in deionized water (1:25, *w/v*). The extraction was performed under shaking in a multi-rotator (Multi RS-60, Biosan, Latvia) at room temperature for 30 min. After centrifugation (3000 rpm, 10 min, Megafuge 16 centrifuge, Heraeus, Hanau, Germany) the supernatants were collected. The residue was re-extracted with 5 mL of deionized water for 15 min. After re-extraction, the samples were centrifuged again and the supernatants were finally combined. Then, 990 µL of the supernatant were mixed with of the internal standard (10 µL norvaline 2 mg/mL) in an injection vial for amino acid analysis. The procedure was carried out in triplicate.

#### 2.3.3. Chromatographic Analysis

The total and free amino acids contents were analyzed by RP-HPLC (Reverse Phase-High Performance Liquid Chromatography) using an integrated system from Jasco (Jasco, Japan) equipped with two high-pressure pumps (PU-980), an automatic injector (AS-4150), a fluorescence detector (FP-2020 Plus), and a UV/Vis absorption detector (MD-2015 Plus). The samples (extracts of total and free amino acids) were derivatized online with OPA/3-MPA and FMOC as described by Machado et al. [25]. Amino acids were identified based on the retention time of the corresponding standards. The quantification of each amino acid was based on the response of the fluorescence signal of each standard, converted into units of concentration through calibration curves obtained for each compound, using the internal standard method. The determination of the total and free amino acid contents was performed in triplicate.

### 2.4. Evaluation of Protein Quality

The AAS and the EAAI were used to assess the protein quality of the analyzed seaweeds. These parameters were calculated as follows (Equations (1) and (2)) [26,27]:

AAS (%) = [mg AA in 1 g of the protein tested/mg AA in 1 g reference protein] × 100(1)

EAAI (%) = *n*^ log EAA (2)

where log EAA = [1/*n*] × [log (100 a1/a1R) + … + log (100 a*n*/a*n*R)]; *a* in mg of amino acid in 1 g of tested protein; *aR* in mg of amino acid in 1 g of reference protein; *n* is the number of amino acids considered for the calculation (the pair methionine-cysteine count as 1). AA and EAA stand for amino acid and essential amino acids in Equations (1) and (2), respectively.

The reference protein used was the amino acid pattern defined by the World Health Organization and the Food and Agriculture Organization (FAO/WHO/UNU) [27]. The AAS of protein was considered the lowest AAS value within essential amino acids.

### 2.5. Determination of Total Nitrogen, Protein Nitrogen, and Non-protein Nitrogen

Total nitrogen (TN) was estimated by Kjeldahl analysis (AOAC, method 984.13) [28]. Protein nitrogen (PN) was determined as described by Machado et al. [25]. Each sample was precipitated with 15% (*w/v*) trichloroacetic acid prior filtering the solution using a low N content filter paper (GPL, Porto, Portugal). The residue was then digested, distilled, and titrated under the above-mentioned conditions.

Non-protein nitrogen (NPN) was estimated from the difference between TN and PN (insoluble nitrogen in 15% (*w/v*) trichloroacetic acid). The protein content was estimated using the conversion factor 5.00 [19]. Analyses were performed in duplicate and the results expressed as g/100 g dried sample (ds).

### 2.6. Statistical Analysis

Statistical analysis was performed using SPSS software, v. 26 (IBM Corporation, Armonk, NY, USA). Data were analyzed by one-way analysis of variance (ANOVA) at a significance level of *p* < 0.05. Where applicable, multiple comparisons were performed using Tukey′s post-hoc test. Data are presented as mean ± standard deviation (SD) of triplicate extractions.

## 3. Results and Discussion

In this study, the total and free amino acid profiles of four seaweed species (*Porphyra dioica*, *Porphyra umbilicalis*, *Gracilaria vermiculophylla,* and *Ulva rigida*) produced in an IMTA system were assessed. Their protein content and protein quality based on the corresponding amino acid score (AAS) and essential amino acid index (EAAI) were also determined.

### 3.1. Amino Acids Composition

#### 3.1.1. Total Amino Acids

The composition in total amino acids of the four different species of seaweeds is presented in Table 1. For both species of *Porphyra* it was possible to quantify the amino acids of the sporophyte (*conchocelis*) and gametophyte (blades) life cycle stages. In general, the analyzed seaweeds presented a similar amino acid profile, although showing significant differences (*p* < 0.05) in the amounts of some compounds.

The sum of total amino acid residues (∑TAA) refers to the true protein content of seaweeds. *Porphyra* species (193.34–203.99 ds and 230.34–286.56 mg/g ds, for blades and *conchocelis*, respectively) showed a significantly higher (*p* < 0.05) ∑TAA compared to *Gracilaria vermiculophylla* (106.62 mg/g ds) and *Ulva rigida* (92.22 mg/g ds). The ∑TAA in the *Porphyra dioica* blades (203.99 ± 8.20 mg/g ds) and *Porphyra umbilicalis* blades (193.34 ± 2.16 mg/g ds) was consistent with the values reported by Biancarosa et al. [29] (242 and 177 mg/g dw, respectively). The lowest levels of ∑TAA were found in *Gracilaria vermiculophylla* and *Ulva rigida* but with no significant (*p* < 0.05) differences between these samples. Higher values have been described by Shuuluka et al. [30] for *Ulva rigida* (152 mg/g dw). The values obtained for *Gracilaria vermiculophylla* ∑TAA were comparable with other species of *Gracilaria*, namely *Gracilaria changii* (91.90 mg/g dw) [31] and *Gracilaria birdiae* (91 mg/g dw) [32]. This may reflect differences in the chemical composition of those samples related with the species itself, harvest season, geographic location, and diverse environmental conditions [33,34].

For the *Porphyra* species, the ∑TAA in the *conchocelis* stage (286.56–230.34 mg/g ds for *Porphyra dioica* and *Porphyra umbilicalis*, respectively) was significantly higher (*p* < 0.05) compared to the corresponding blades stage (203.99–193.34 mg/g ds, by the same order). As described for phycobiliproteins [35], the higher content of amino acids in the *conchocelis* stage might be related to the pathways of nitrogen uptake, utilization, and storage. It should also be pointed out that cell walls present a different chemical structure at the different life cycle stages. In blades, these present more than twice of the fiber content compared to that found in *conchocelis*. Hence, the latter are reported to present significantly higher protein values compared to blades [36].

#### 3.1.2. Essential Amino Acids

Despite the quantitative differences observed between the different species for the individual compounds, all samples presented a complete profile of EAA (Table 1). The most abundant EAA in red species (*Porphyra dioica*, *Porphyra umbilicalis,* and *Gracilaria vermiculophylla*) were leucine, valine, and threonine. The leucine values ranged from 9.01 to 16.63 mg/g ds; valine from 6.84 to 12.79 mg/g ds; and threonine from 6.21 to 12.27 mg/g ds. Significant differences were observed (*p* < 0.05) between the *Porphyra* species and *Gracilaria vermiculophylla*, which presented the lowest values for the aforementioned compounds. These results are consistent with other studies [33,37,38]. The amino acid composition of proteins is frequently used to determine its nutritional quality [6]. High quality protein presents a complete and optimal EAA composition for human needs. Most dietary protein of animal origin (meat, fish, milk, and egg) present higher levels of EAA being considered as high quality protein [39,40]. In contrast, some plant-based dietary proteins can be seen as being of lower nutritional quality due to their low content in one or several EAA [40]. EAA have important roles in the body, namely branched-chain amino acids, which are essential for the proliferation of lymphocytes and maturation of dendritic cells, and some inhibitory effect in cancer cell proliferation [41]. From another perspective, the incorporation of *Porphyra umbilicalis* in pork products promoted a significant increase in serine, glycine, alanine, valine, tyrosine, phenylalanine, and arginine levels of the formulations [42]. The high lysine concentrations found in the analyzed samples may be interesting, as seaweeds may be used to balance the amino acids composition of cereal-based products, which often contain a low lysine content, also considered the limiting amino acid in plant-based proteins [43].

*Porphyra* spp. samples presented a similar EAA profile. However, *Porphyra umbilicalis* blades had a significantly higher (*p* < 0.05) tryptophan content (0.79 ± 0.03 mg/g ds) compared to that of *Porphyra dioica* (0.59 ± 0.03 mg/dry ds). *Conchocelis* presented significantly higher (*p* < 0.05) levels of EAA when compared with the corresponding blades. *Porphyra dioica conchocelis* showed significantly higher levels (*p* < 0.05) of all EAA. The most abundant EAA in this sample were lysine, leucine, and valine (22.33 ± 0.35, 22.03 ± 0.25 and 18.24 ± 0.25 mg/g ds, respectively). *Porphyra umbilicalis conchocelis* presented significantly higher levels (*p* < 0.05) of histidine, methionine, and tryptophan compared to blades of the same species. The EAA in larger quantities in *Porphyra umbilicalis conchocelis* were also leucine, lysine, and valine (17.32 ± 1.03, 16.08 ± 0.68 and 13.63 ± 0.84 mg/g ds, respectively). In *Ulva rigida*, the major essential amino acids were leucine, phenylalanine, and valine (7.89 ± 0.09, 5.79 ± 0.03, 5.78 ± 0.03 mg/g ds, respectively).

Overall, tryptophan, methionine, and histidine presented the lower EAA levels in all analyzed samples. These results are also consistent with previously reported data [33,44,45,46]. However, it should be noted that *Ulva rigida* had a significantly higher (*p* < 0.05) histidine content (2.96 ± 0.07) compared with the other analyzed species in their adult stage: *Gracilaria vermiculophylla* (1.14 ± 0.04 mg/g ds), *Porphyra umbilicalis* (1.66 ± 0.05 mg/g ds), and *Porphyra dioica* (2.05 ± 0.50 mg/g ds).

The percentage in essential amino acids (% EAA) of *Ulva rigida* (40.79 ± 0.23%) was significantly higher (*p* < 0.05) than both species of *Porphyra*. Histidine content of *Porphyra umbilicalis* was similar (*p* < 0.05) between the two life cycle stages (38.45 ± 0.10 and 38.65 ± 0.28% for blades and *conchocelis*, respectively). The % EAA in *Gracilaria vermiculophylla* was similar to *Ulva rigida* (40.14 ± 0.09 and 40.79 ± 0.23%, respectively).

The % EAA for *Porphyra dioica* and *Porphyra umbilicalis* blades was similar to the values presented by Biancarosa et al. [29] (38.7 and 38.5% dry weight (dw), respectively). However, the values obtained herein were lower than those presented by Vieira et al. [8] (39.9–44.3% dw for *Porphyra* spp). The % EAA presented for *Ulva rigida* was higher than that presented by Shuuluka et al. [28] (30.8% dw), and comparable to what Lourenço et al. [47] have reported for *Ulva fasciata* (41.4% dw). The % EAA in *Gracilaria vermiculophylla* was lower than the values previously described for other species of *Gracilaria*, which ranged from 50–61% dw [31,32].

These results indicate that the seaweed species analyzed in this study present similar amounts of EAA compared to other plant-based protein sources, such as lupine (38.01% dw), faba bean (41.36% dw), hemp (39.52% dw), and flaxseed (38.82% dw) [48], or animal origin, as is the case with casein (43.6%), although lower than those of ovalbumin (52.4%) [34].

#### 3.1.3. Non-Essential Amino Acids

Acid aspartic was the most abundant non-essential amino acid (NEAA) in all samples, ranging from 12.05 to 24.15 mg/g ds in adult seaweeds, reaching 32.49 ± 0.21 mg/g ds in *Porphyra dioica conchocelis*. Glutamic acid was the second most abundant amino acid in all species ranging from 9.47 to 31.48 mg/g ds, except in blade stage of *Porphyra* species, which was richer in alanine. The content of acidic amino acids ranged from 22.33 ± 0.07% to 23.60 ± 0.28% of the total amino acids obtained in *Porphyra dioica conchocelis* and in *Gracilaria vermiculophylla*, respectively. Astorga-España et al. [33] also found high levels of these amino acids for *Porphyra* spp. and *Ulva* spp. (22.98 and 21.79%, respectively). The levels of acidic amino acids include the simultaneous quantification of the amides (glutamine and asparagine), which are converted to the corresponding acids during acid hydrolysis [45].

All samples have also presented high concentrations of other NEAA such as alanine, arginine, serine, and glycine. The presence of these amino acids is interesting due to their important biological functions. For instance, arginine supplementation improves the function of the intestinal barrier and vascular development [49]; glycine helps in the regulation of the immune response and helps to prevent rejections after organ transplantation [50]; alanine is used to treat muscle degeneration [9]; serine supplementation may be useful in the treatment of hereditary sensory and autonomic neuropathy type 1 [51]. From another perspective, high levels of glutamic acid, aspartic acid, alanine, and glycine have been described as responsible for the characteristic umami flavor of seaweeds [34,45]. Hydroxyproline was the NEAA that presented the lowest concentration, reaching the maximum of 1.06 ± 0.02 mg/g ds in the green algae *Ulva rigida*. All other samples presented hydroxyproline values ≤0.26 mg/g ds. *Porphyra* blades had a similar profile of NEAA (*p* > 0.05). Significant differences were only observed in glycine content (16.75 ± 0.74 and 13.61 ± 0.09 ±, for *Porphyra dioica* and *Porphyra umbilicalis* blades, respectively). Overall, *Porphyra dioica* blades had higher levels of NEAA (*p* < 0.05) than those found in *Porphyra umbilicalis* (63.16 ± 0.44 and 61.55 ± 0.10 mg/g ds). *Porphyra dioica conchocelis* had a significantly higher (*p* < 0.05) content of aspartic acid, glutamic acid, serine, arginine, alanine, tyrosine (32.49 ± 0.21 > 31.48 ± 0.43 > 30.25 ± 0.29 > 23.68 ± 0.24 > 16.42 ± 0.20 > 10.17 ± 0.16 mg/g ds, respectively) than the blades and *conchocelis* of *Porphyra umbilicalis*. Proline content was similar in both stages of the analyzed *Porphyra* species.

#### 3.1.4. Free Amino Acids

Free amino acids are not linked to other amino acids, peptides, or proteins, usually contributing to the food taste [6]. These amino acids act as one of the main reservoirs for storing nitrogen in both green and red seaweeds [52].

The free amino acid composition of the four different seaweeds species, including the two life cycle stages of *Porphyra*, is shown in Table 2 in which it is possible to observe significant differences (*p* < 0.05) in the amino acid composition between the analyzed species, even between the life stages of the same *Porphyra* species.

The sum of free amino acids represents 7.18, 6.65, 5.73, 5.64, 5.14, and 3.15% of the total amino acids fraction in the *Porphyra umbilicalis* blades, *Porphyra dioica* blades, *Porphyra umbilicalis conchocelis, Porphyra dioica conchocelis, Ulva rigida,* and *Gracilaria vermiculophylla*, respectively. These values are within the range (3.40–14.00%) reported by Vieira et al. [8] for *Gracilaria* spp., *Porphyra* spp., and *Ulva* spp. For *Porphyra dioica conchocelis* (16.17 ± 0.03 mg/g ds) the sum of free amino acids was significantly higher (*p* < 0.05) compared to the other seaweeds analyzed, similarly to what was previously observed for its total amino acids.

The free amino acids composition was mainly represented by alanine, glutamic acid, and aspartic acid in both *Porphyra* species and *Gracilaria Vermiculophylla*, presenting concentrations ranging from 0.26 to 5.50, 1.14 to 4.77, and 0.53 to 3.53 mg/g ds, respectively. These amino acids play an important role in the umami flavor, characteristic of seaweeds [45]. Noda et al. [53] also found that the predominant free amino acids in *Porphyra* spp. were aspartic acid, glutamic acid, alanine, and taurine. Admassu et al. [54] found that in addition to the above-mentioned amino acids, arginine was also one of the main amino acids in *Porphyra* spp. In *Ulva rigida*, the most abundant amino acid was histidine (1.96 ± 0.09 mg/g ds) and asparagine (1.45 ± 0.06 mg/g ds), presenting significantly higher (*p* < 0.05) concentrations compared to the remaining seaweeds. Methionine and tryptophan were only detected in *Porphyra dioica conchocelis*. In the red seaweeds species, the free amino acid composition was represented in over 80% by NEAA. *Ulva rigida* presented a different proportion between EAA and NEAA, corresponding to approximately 45 and 55%, respectively.

### 3.2. Evaluation of Protein Quality Based on the Amino Acids Profile

The composition, proportion, and availability of essential amino acids in proteins contribute to determine the nutritional quality of food [33]. The EAA/NEAA ratio was used to evaluate the distribution of those amino acids in proteins from the analyzed seaweeds. The results ranged from 0.58 to 0.67 within the red species and reached 0.69 in the green algae *Ulva rigida*. These values indicate that EAA were in lower concentrations compared to NEAA in all samples. Higher values have reported for *Gracilaria* spp., *Porphyra* spp. and *Ulva* spp., which have presented an EAA/NEAA ratio of 1.74, 1.32, and 1.32, respectively [8] determined an EAA/NEAA ratio of 1.74, 1.32, and 1.32 for the *Gracilaria* sp., *Porphyra* sp., and *Ulva* sp., respectively. Nevertheless, the results obtained herein are similar to those reported by other authors: 0.63 in both *Porphyra umbilicalis* and *Porphyra dioica* [29]; 0.54 and 0.56 for *Porphyra* spp. and *Ulva* spp. [33]. Once again, several factors (seasonality, place of production...) influence the composition of seaweeds, namely their composition in amino acids, which may be the cause of the differences observed [29].

In this study, two important chemical parameters were used to evaluate protein quality: the AAS and the EAAI. The first is intended to predict protein quality relying on the potential ability of a food protein to provide the appropriate pattern of dietary essential amino acids [55]. The EAAI compares the protein quality through the geometric mean value of the essential amino acid in relation to a reference protein [37]. EAAI reflects more the biological quality of a protein than AAS [37]. Table 3 presents the AAS and EAAI values for all samples, based on the recommended amino acid pattern for adults, according to FAO/WHO/UNU [27]. As tyrosine can substitute phenylalanine, through metabolic processes, these amino acids were combined (tyrosine + phenylalanine) for the calculation of AAS [33]. From the AAS it was possible to determine the limiting amino acid, which corresponds to the essential amino acid that presented the greatest difference in concentration compared with the same amino acid in the reference protein [8].

The AAS was 48.7% for *Porphyra dioica* blades, 56.5% for *Porphyra umbilicalis* blades, 58.4% for *Gracilaria vermiculophylla*, and *90.8*% for *Ulva rigida*. *Conchocelis* had a higher AAS than the corresponding blades (87.1 and 93.4%, for *Porphyra dioica* and *Porphyra umbilicalis*, respectively). The concentrations of isoleucine, leucine, lysine, threonine, phenylalanine + tyrosine were higher than the FAO/WHO/UNU standard [27] for all samples, meaning that the corresponding AAS values exceeded 100%. Methionine and tryptophan were the first limiting amino acids in all red seaweeds species. For *Porphyra umbilicalis* blades histidine was the second limiting amino acid. These results are in agreement with other authors [8,31,34,37] which reported that sulfur-containing amino acids, tryptophan, and histidine are the main limiting amino acids. Benjama and Masniyom [56] and Mišurcová et al. [45] found lysine as the main limiting amino acid in *Gracilaria* and *Porphyra*, with AAS ranging from 41.6 to 82.2% in *Gracilaria*. However, AAS values obtained herein for lysine were relatively high, ranging from 109.72% to 173.20% for the *Ulva rigida* and *Porphyra dioica conchocelis*, respectively. These differences may be related to the reference protein used, species, geographical origin, season of harvesting, environmental conditions, and the physiology of each species [33]. The EAAI ranged from 90.77% to 115.74% in red species, reaching 123.38% in *Ulva rigida*. The protein quality of the *Ulva rigida* exceeded that of the other seaweeds, that is, it exhibited an amino acid profile closer to the reference protein. A protein with high quality and efficiency is generally characterized by a high EAAI value. According to Brown and Jeffrey [57], a protein has high quality when the EAAI value is greater than 90%, moderate quality when the EAAI is between 70–89% and low quality when the EAAI is less than 70%. Based on the reference standard [27], the proteins of the analyzed seaweeds can be considered of high quality. Therefore, based on the results, these seaweed species could be used as a source of high quality protein, or as ingredients to improve the amino acid profile of food formulations.

### 3.3. Nitrogen and Protein Content

Three different approaches were used to assess the protein content samples, which comprised: (i) the conversion of total nitrogen (determined by the Kjeldahl method) to protein by the conversion factor 5.00 [19]; (ii) the conversion of protein nitrogen (following precipitation with trichloroacetic acid) to protein using the conversion factor 5.00 [19]; (iii) the sum of the total amino acids. Method (i) estimates the crude protein, while the latter (ii and iii) estimates the “true protein.” Table 4 shows the nitrogen content (non-protein, protein, and total) and the estimated protein content based on the above-mentioned approaches. The main disadvantage of the Kjeldahl method is the quantification of non-protein nitrogen, which includes chlorophyll, nucleic acids, free amino acids, and inorganic nitrogen [19]. The use of the universal nitrogen-to-protein conversion factor of 5.00, proposed by Angell et al. [19] allows for estimating the protein content of seaweeds more accurately, based on the protein nitrogen fraction. However, it should be noted that this proposed factor is a median value and that seaweeds have a variable amino acid composition and amount of non-protein nitrogen. The protein content estimation according to approach (iii) is widely accepted since the 1970s, although it presents some disadvantages [33].

Amino acid analysis can underestimate protein content due to partial or total destruction of some amino acids during hydrolysis (in particular, cysteine, tryptophan, methionine, and serine); furthermore, the use of a single hydrolysis time may not assure the complete hydrolysis of certain amino acids without destroying others [19]. However, Lourenço et al. [47] stated that if a sample contains 10% free amino acids, the typical loss during acid hydrolysis might compensate for the influence of free amino acids in the protein quantification using the sum of the total amino acid residues. In this study, the free fraction was less than 10% of the total amino acids, thus there might have occurred an underestimation when assessing the true protein values using the sum of total amino acid residues.

In general, the genus *Porphyra* had a significantly higher (*p* < 0.05) protein content compared to *Gracilaria vermiculophylla* and *Ulva rigida*, regardless of the approach used. The crude protein content was slightly higher than the true protein content (estimated by the conversion NP × 5.00, and obtained by the ∑TAA), except for *Porphyra dioica conchocelis*. Notwithstanding, the obtained values were within a similar range.

*Ulva rigida* showed a significantly lower (*p* < 0.05) protein nitrogen content (0.22 ± 0.17 g/100 g ds) compared with the *Porphyra dioica* blades (1.12 ± 0.04 g/100 g ds). No significant differences (*p* > 0.05) were observed for the protein nitrogen content of the red seaweed species. In fact, Lourenço et al. [47] reported that the latter present higher amounts of protein nitrogen than green and brown seaweeds, corroborating the results obtained herein.

The protein content of the analyzed seaweeds (9.62–28.66 g/100 g ds) was comparable to that of protein-rich plant-based foods, such as beans (20.9%), lupine (30.5%), chickpeas (24.7%), linseed (26.35%), peanuts (29.59%), and rice (9.57%) [43]. The used algal species have great potential to be used in human nutrition as a source of protein. However, the digestibility of these proteins must be evaluated, as it tends to be limited by the non-protein fraction [37].

There are other concerns regarding the consumption of seaweeds, namely those related with the excessive intake of iodine. The recommended daily allowance (RDA) by the World Health Organization is 150 µg for adults [58]. A small amount of seaweeds can exceed the tolerable intake limit for humans (1100 µg), and consequently affect thyroid health [6]. For example, a portion of 0.3 g (dw) of *Laminaria* spp. may exceed the tolerable intake limit. On the other hand, it is reported that 5 g (dw) of *Porphyra tenera* or *Ulva rigida* contain 80 µg (53% of RDA) and 40 µg (27% of RDA) of iodine, respectively [6], is what can be considered as safe. The concentration of iodine in different seaweed species is highly variable; therefore, the content of this micronutrient must be accurately labelled to avoid excessive intake [6]. Generally, *Porphyra* and *Ulva* species have lower levels of iodine compared to brown algae [6]. The consumption of a portion of 5 g (dw) of *Porphyra* or *Ulva* may be the most adequate, in order to maintain a safety margin. However, elements were not analyzed in this study.

Based on the results of this study, the consumption of a 5 g portion (ds) of *Porphyra dioica conchocelis, Porphyra umbilicalis conchocelis, Porphyra dioica* blades, *Porphyra umbilicalis* blades, *Gracilaria vermiculophylla*, and *Ulva rigida* provides an average of 1.43, 1.15, 1.02, 0,97, 0.53, and 0.48 g of protein, respectively. Considering that the RDA of protein intake for an adult of 70 kg is equivalent to 58 g [25] the intake of 5 g (ds) of the *Porphyra dioica conchocelis, Porphyra umbilicalis conchocelis, Porphyra dioica* blades, *Porphyra umbilicalis* blades, *Gracilaria vermiculophylla*, and *Ulva rigida* contributes to 2.47, 1.99, 1.76, 1.67, 0.92, and 0.83% of RDA, respectively. Based on these observations, seaweeds can be a sustainable alternative to diversify or complement the diet. Extracting proteins from seaweeds may also be a strategy to take advantage of their high protein content, as the presence of iodine or even heavy metals restricts the intake of larger seaweed portions. Seaweed-derived protein isolates or hydrolysates may be used as food ingredients, contributing, for example, to improve the composition of high protein food formulations. The inclusion of seaweeds (protein extracts) in food products can bring added-value for the food industry. However, efforts are needed in the food biotechnology field in order to make this process viable and accessible.

## 4. Conclusions

Results obtained in this study demonstrated that the analyzed seaweeds, which were produced in an IMTA system, presented a complete EAA profile and, consequently, a high quality protein profile, according to the FAO/WHO/UNU standards. *Porphyra* species (both life cycle stages) were characterized by a higher protein content compared to *Gracilaria vermiculophylla* and *Ulva rigida*. *Conchocelis*, in particular, had the highest protein content. As the free amino acid fraction in red seaweeds was characterized by a high content in the alanine, glutamic acid, and aspartic acid (amino acids responsible for umami flavor), these may possibly be extracted and used as flavor enhancers. These results highlight the potential of using seaweeds as an alternative and sustainable source of protein and amino acids for human nutrition and industrial food processing.

Overall, it appears that the production of these seaweed species in an IMTA system contributed to the production of high protein quality biomass.

## Figures and Tables

**Table 1:** Total amino acids (TAA) composition expressed in mg/g of dry sample (ds) of *Porphyra dioica* (blades and *conchocelis*), *Porphyra umbilicalis* (blades and *conchocelis*), *Gracilaria vermiculophylla,* and *Ulva rigida.*

Amino Acids	*Porphyra dioica*		*Porphyra umbilicalis*		*Gracilaria vermiculophylla* (mg/g ds)	*Ulva rigida* (mg/g ds)
	Blades (mg/g ds)	*Conchocelis* (mg/g ds)	Blades (mg/g ds)	*Conchocelis* (mg/g ds)		
Asp	24.15 ± 0.64 ^bc^	32.49 ± 0.21 ^a^	23.69 ± 0.65 ^c^	26.61 ± 1.75 ^b^	12.68 ± 0.41 ^d^	12.05 ± 0.29 ^d^
Glu	22.57 ± 0.72 ^c^	31.48 ± 0.43 ^a^	21.07 ± 0.22 ^c^	25.91 ± 1.63 ^b^	12.47 ± 0.54 ^d^	9.47 ± 0.23 ^e^
Ala	23.30 ± 0.79 ^bc^	30.25 ± 0.29 ^a^	21.76 ± 0.17 ^b^	23.52 ± 1.45 ^b^	8.11 ± 0.35 ^c^	8.48 ± 0.10 ^c^
Arg	14.58 ± 0.54 ^c^	23.68 ± 0.24 ^a^	14.27 ± 0.11 ^c^	17.88 ± 1.23 ^b^	8.37 ± 0.35 ^d^	6.06 ± 0.07 ^e^
Gly	16.75 ± 0.74 ^a^	18.22 ± 0.22 ^a^	13.61 ± 0.09 ^b^	16.65 ± 0.93 ^a^	6.82 ± 0.36 ^c^	6.67 ± 0.65 ^c^
Ser	12.05 ± 0.44 ^bc^	16.42 ± 0.20 ^a^	10.54 ± 0.12 ^c^	13.26 ± 0.80 ^b^	6.75 ± 0.22 ^d^	5.54 ± 0.06 ^d^
Tyr	6.15 ± 0.25^c^	10.17 ± 0.16 ^a^	5.47 ± 0.09 ^c^	8.75 ± 0.59 ^b^	3.53 ± 0.13 ^d^	3.25 ± 0.07 ^d^
Pro	9.08 ± 0.32 ^a^	9.79 ± 0.32 ^a^	8.60 ± 0.08 ^a^	8.73 ± 0.71 ^a^	4.82 ± 0.18 ^b^	4.40 ± 0.10 ^b^
Hyp	0.15 ± 0.01 ^c^	0.05 ± 0.01 ^d^	n.d.	0.05 ± <0.01 ^d^	0.26 ± 0.01 ^b^	1.06 ± 0.02 ^a^
Phe	9.27 ± 0.35 ^b^	11.68 ± 0.07 ^a^	8.56 ± 0.07 ^b^	9.12 ± 0.58 ^b^	6.15 ± 0.30 ^c^	5.79 ± 0.03 ^c^
His	2.05 ± 0.50 ^c^	6.62 ± 0.08 ^a^	1.66 ± 0.05 ^cd^	6.46 ± 0.29 ^a^	1.14 ± 0.04 ^d^	2.96 ± 0.07 ^b^
Ile	8.22 ± 0.34 ^b^	11.29 ± 0.17 ^a^	8.31 ± 0.07 ^b^	8.42 ± 0.53 ^b^	5.86 ± 0.28 ^c^	4.44 ± 0.06 ^d^
Leu	16.63 ± 0.62 ^b^	22.03 ± 0.25 ^a^	16.12 ± 0.15 ^b^	17.32 ± 1.03 ^b^	9.01 ± 0.39 ^c^	7.89 ± 0.09 ^c^
Lys	11.60 ± 1.15 ^c^	22.33 ± 0.35 ^a^	11.58 ± 0.19 ^c^	16.08 ± 0.68 ^b^	5.80 ± 0.19 ^d^	4.76 ± 0.70 ^d^
Met	2.22 ± 0.24 ^c^	5.49 ± 0.05 ^a^	2.40 ± 0.04 ^c^	4.73 ± 0.28 ^b^	1.37 ± 0.07 ^d^	1.92 ± 0.05 ^c^
Thr	12.27 ± 0.34 ^b^	14.82 ± 0.15 ^a^	12.13 ± 0.09 ^b^	11.87 ± 0.66 ^b^	6.21 ± 0.24 ^c^	4.88 ± 0.14 ^d^
Trp	0.59 ± 0.03 ^d^	1.50 ± 0.05 ^a^	0.79 ± 0.03 ^c^	1.37 ± 0.02 ^b^	0.42 ± 0.01 ^e^	0.85 ± 0.02 ^c^
Val	12.34 ± 0.42 ^b^	18.24 ± 0.25 ^a^	12.79 ± 0.09 ^b^	13.63 ± 0.84 ^b^	6.84 ± 0.35 ^c^	5.78 ± 0.03 ^c^
∑TAA	203.99 ± 8.20 ^c^	286.56 ± 3.11 ^a^	193.34 ± 2.16 ^c^	230.34 ± 13.79 ^b^	106.62 ± 4.09 ^d^	96.22 ± 0.78 ^d^
% EAA	36.84 ± 0.44 ^d^	39.78 ± 0.06 ^b^	38.45 ± 0.10 ^c^	38.65 ± 0.28 ^c^	40.14 ± 0.09 ^ab^	40.79 ± 0.23 ^a^
% NEAA	63.16 ± 0.44 ^a^	60.22 ± 0.06 ^c^	61.55 ± 0.10 ^b^	61.35 ± 0.28 ^b^	59.86 ± 0.09 ^cd^	59.21 ± 0.23 ^d^
EAA/NEAA	0.58 ± 0.01 ^d^	0.66 ± <0.01 ^b^	0.62 ± <0.01 ^c^	0.63 ± 0.01 ^c^	0.67 ± <0.01 ^ab^	0.69 ± 0.01 ^a^

Amino acids are represented by the 3-letter abbreviation code. In each row, different superscript letters represent significant differences between samples (*p* < 0.05), while the same superscript letters denote no significant differences (*p* > 0.05). n.d.: not detected.

**Table 2:** Free amino acids composition expressed in mg/g of dry sample (ds) of *Porphyra dioica* (blades and *conchocelis*), *Porphyra umbilicalis* (blades and *conchocelis*), *Gracilaria vermiculophylla,* and *Ulva rigida.*

Amino Acids	*Porphyra dioica*		*Porphyra umbilicalis*		*Gracilaria vermiculophylla* (mg/g ds)	*Ulva rigida* (mg/g ds)
	Blades (mg/g ds)	*Conchocelis* (mg/g ds)	Blades (mg/g ds)	*Conchocelis* (mg/g ds)		
Asp	2.23 ± 0.01 ^b^	1.30 ± 0.01 ^c^	3.53 ± 0.09 ^a^	1.44 ± 0.09 ^c^	0.53 ± 0.03 ^d^	0.24 ± <0.01 ^e^
Glu	3.67 ± 0.05 ^b^	4.63 ± 0.03 ^a^	3.15 ± 0.08 ^c^	4.77 ± 0.15 ^a^	1.14 ± 0.03 ^d^	0.21 ± 0.01 ^e^
Asn	0.55 ± 0.01 ^b^	0.35 ± <0.01 ^d^	0.44 ±0.01 ^c^	0.32 ± 0.02 ^d^	0.14 ± <0.01 ^e^	1.45 ± 0.06 ^a^
Gln	0.09 ± <0.01 ^d^	0.33 ± <0.01 ^a^	0.14 ± <0.01 ^b^	0.13 ± 0.01 ^c^	0.08 ± <0.01 ^e^	0.08 ± <0.01 ^de^
Ala	5.50 ± 0.06 ^a^	5.37 ± 0.04 ^a^	5.01 ± 0.08 ^b^	3.70 ± 0.13 ^c^	0.26 ± 0.01 ^d^	0.19 ± 0.01 ^d^
Arg	0.12 ± 0.01 ^c^	0.70 ± 0.01 ^a^	0.14 ± <0.01 ^c^	0.29 ± 0.01 ^b^	0.13 ± 0.02 ^c^	0.08 ± <0.01 ^d^
Gly	0.13 ± 0.01 ^d^	0.37 ± <0.01 ^a^	0.15 ± 0.01 ^cd^	0.29 ± 0.05 ^b^	0.10 ± 0.01 ^d^	0.20 ± 0.01 ^c^
Ser	0.26 ± <0.01 ^c^	0.45 ± 0.01 ^b^	0.29 ± <0.01 ^c^	0.77 ± 0.03 ^a^	0.19 ± 0.01 ^d^	0.13 ± <0.01 ^e^
Tyr	0.04 ± <0.01 ^c^	0.18 ± <0.01 ^a^	0.07 ± 0.01 ^b^	0.05 ± <0.01 ^c^	0.07 ± <0.01 ^b^	0.07 ± <0.01 ^b^
Pro	0.04 ± <0.01 ^de^	0.14 ± 0.01 ^a^	0.03 ± <0.01 ^e^	0.12 ± 0.01 ^b^	0.05 ± 0.01 ^d^	0.07 ± <0.01 ^c^
Hyp	0.01 ± <0.01 ^b^	n.d.	0.01 ± <0.01 ^b^	n.d.	0.01 ± <0.01 ^a^	0.01 ± <0.01 ^a^
Phe	0.05 ± <0.01 ^d^	0.27 ± <0.01 ^a^	0.08 ± <0.01 ^c^	0.11 ± <0.01 ^b^	0.05 ± <0.01 ^d^	0.04 ± <0.01 ^d^
His	0.18 ± 0.01 ^b^	0.06 ± <0.01 ^c^	0.02 ± <0.01 ^c^	0.03 ± 0.01 ^c^	0.20 ± 0.02 ^b^	1.96 ± 0.09 ^a^
Ile	0.07 ± <0.01 ^b^	0.17 ± <0.01 ^a^	0.07 ± <0.01 ^b^	0.08 ± <0.01 ^b^	0.05 ± <0.01 ^c^	0.04 ± <0.01 ^c^
Leu	0.07 ± <0.01 ^c^	0.39 ± <0.01 ^a^	0.07 ± <0.01 ^c^	0.13 ± 0.01 ^b^	0.04 ± <0.01 ^d^	0.05 ± <0.01 ^d^
Lys	0.11 ± <0.01 ^cd^	0.37 ± 0.01 ^a^	0.13 ± <0.01 ^c^	0.25 ± 0.03 ^b^	0.10 ± 0.01 ^cd^	0.07 ± <0.01 ^d^
Met	n.d.	0.16 ± <0.01	n.d.	n.d.	n.d.	n.d.
Thr	0.28 ± <0.01 ^d^	0.46 ± 0.01 ^b^	0.41 ± 0.01 ^c^	0.53 ± 0.03 ^a^	0.16 ± 0.01 ^e^	n.d.
Trp	n.d.	0.09 ± <0.01	n.d.	n.d.	n.d.	n.d.
Val	0.16 ± 0.01 ^c^	0.37 ± <0.01 ^a^	0.14 ± <0.01 ^c^	0.21 ± 0.01 ^b^	0.08 ± 0.01 ^d^	0.06 ± <0.01 ^e^
∑FAA	13.57 ± 0.15 ^bc^	16.17 ± 0.03 ^a^	13.88 ± 0.28 ^b^	13.20 ± 0.23 ^c^	3.36 ± 0.11 ^e^	4.95 ± 0.15 ^d^

FAA: Free amino acids; ds: dry sample. Amino acids are presented by the 3-letter abbreviation code. In each row, different superscript letters represent significant differences between samples (*p* < 0.05) while the same superscript letters denote no significant differences (*p* > 0.05). n.d.: not detected.

**Table 3:** Amino acid score (AAS) and essential amino acid index (EAAI) values for *Porphyra dioica* (blades and *conchocelis*), *Porphyra umbilicalis* (blades and *conchocelis*), *Gracilaria vermiculophylla,* and *Ulva rigida*.

Essential Amino Acids	Amino Acids Scoring Pattern	*Porphyra dioica*		*Porphyra umbilicalis*		*Gracilaria vermiculophylla*	*Ulva rigida*
	(mg AA/g Protein) [27]	Blades	*Conchocelis*	Blades	*Conchocelis*		
His	15	9.96 ± 2.61 ^c^	23.09 ± 0.11 ^b^	8.56 ± 0.19 ^c^	28.05 ± 0.67 ^a^	10.70 ± 0.29 ^c^	30.71 ± 0.64 ^a^
Ile	30	40.30 ± 0.07 ^d^	39.39 ± 0.22 ^d^	42.99 ± 0.22 ^c^	36.55 ± 0.15 ^e^	54.94 ± 0.66 ^a^	46.11 ± 0.87 ^b^
Leu	59	81.52 ± 0.30 ^b^	76.86 ± 0.05 ^cd^	83.40 ± 0.22 ^ab^	75.21 ± 0.32 ^c^	84.53 ± 0.52 ^a^	82.02 ± 1.71 ^b^
Lys	45	56.76 ± 4.36 ^c^	77.94 ± 0.81 ^a^	59.88 ± 0.58 ^bc^	69.92 ± 2.93 ^ab^	54.40 ± 0.36 ^c^	49.37 ± 8.47 ^c^
Met	22 *	10.87 ± 1.05 ^b^	19.17 ± 0.09 ^a^	12.43 ± 0.22 ^b^	20.54 ± 0.21 ^a^	12.88 ± 1.33 ^b^	19.93 ± 0.77 ^a^
Phe + Tyr	38	75.61 ± 0.14 ^d^	76.24 ± 0.23 ^cd^	72.60 ± 0.27 ^e^	77.53 ± 0.59 ^c^	90.81 ± 0.57 ^b^	93.90 ± 1.09 ^a^
Thr	23	60.17 ± 0.96 ^ab^	51.71 ± 0.10 ^cd^	62.73 ± 0.42 ^a^	51.54 ± 0.53 ^c^	58.24 ± 0.23 ^b^	50.78 ± 2.21 ^c^
Trp	6	2.92 ± 0.25 ^d^	5.23 ± 0.27 ^b^	4.09 ± 0.26 ^c^	5.96 ± 0.35 ^b^	3.97 ± 0.17 ^c^	8.80 ± 0.37 ^a^
Val	39	60.50 ± 0.47 ^c^	63.65 ± 0.39 ^b^	66.14 ± 0.32 ^a^	59.16 ± 0.65 ^c^	64.09 ± 1.01 ^b^	60.06 ± 0.85 ^c^
LAA	-	Trp	Met	Met	Met	Met	Met
AAS (%)	-	48.7	87.1	56.5	93.4	58.4	90.8
EAAI (%)	-	90.8	114.2	96.5	115.7	101.9	123.4

LAA: limiting amino acid; AAS: amino acid score; EAAI: essential amino acid index. * Met + Cys. Amino acids represented using the 3-letter abbreviation code. In each row, different superscript letters represent significant differences between samples (*p* < 0.05) while the same superscript letters denote no significant differences (*p* > 0.05).

**Table 4:** Nitrogen (non-protein, protein, total), crude and true protein contents for *Porphyra dioica* (blades and *conchocelis*), *Porphyra umbilicalis* (blades and *conchocelis*), *Gracilaria vermiculophylla,* and *Ulva rigida*.

Nitrogen/Protein	*Porphyra dioica*		*Porphyra umbilicalis*		*Gracilaria vermiculophylla* (% ds)	*Ulva rigida* (% ds)
	Blades (% ds)	*Conchocelis* (% ds)	Blades (% ds)	*Conchocelis* (% ds)		
NPN	1.12 ± 0.04 ^a^	0.88 ± 0.01 ^ab^	0.64 ± 0.01 ^ab^	0.46 ± 0.14 ^ab^	0.44 ± 0.23 ^ab^	0.22 ± 0.17 ^b^
PN	3.62 ± 0.01 ^c^	4.47 ± <0.01 ^a^	3.99 ± 0.01 ^bc^	4.33 ± 0.14 ^ab^	2.24 ± <0.01 ^d^	1.82 ± 0.14 ^d^
TN	4.74 ± 0.05 ^b^	5.35 ± 0.01 ^a^	4.62 ± <0.01 ^b^	4.79 ± <0.01 ^b^	2.68 ± 0.23 ^c^	2.04 ± 0.03 ^d^
Crude protein	23.70 ± 0.26 ^b^	26.73 ± 0.03 ^a^	23.11 ± <0.01 ^b^	23.97 ± 0.01 ^b^	13.38 ± 1.16 ^c^	10.19 ± 0.16 ^d^
True protein (NP × 5)	18.10 ± 0.05 ^c^	22.34 ± 0.02 ^a^	19.93 ± 0.03 ^bc^	21.67 ± 0.71 ^ab^	11.19 ± <0.01 ^d^	9.08 ± 0.71 ^d^
True protein (∑AAT)	20.40 ± 0.82 ^c^	28.66 ± 0.31 ^a^	19.33 ± 0.22 ^c^	23.03 ± 1.38 ^b^	10.66 ± 0.41 ^d^	9.62 ± 0.08 ^d^

ds: dry sample; NPN: non-protein nitrogen; PN: protein nitrogen; TN: total nitrogen; Σ AAT: sum of total amino acids. In each row, different letters represent significant differences (*p* < 0.05) between the samples.
